# Plasma Metabolite Signatures in Male Carriers of Genetic Variants Associated with Non-Alcoholic Fatty Liver Disease

**DOI:** 10.3390/metabo13020267

**Published:** 2023-02-13

**Authors:** Lilian Fernandes Silva, Jagadish Vangipurapu, Anniina Oravilahti, Ville Männistö, Markku Laakso

**Affiliations:** 1 Institute of Clinical Medicine, Internal Medicine, University of Eastern Finland, 70210 Kuopio, Finland; 2 Department of Medicine, Kuopio University Hospital, 70210 Kuopio, Finland

**Keywords:** non-alcoholic fatty liver disease, genetic variants, metabolomics

## Abstract

Both genetic and non-genetic factors are important in the pathophysiology of non-alcoholic fatty liver disease (NAFLD). The aim of our study was to identify novel metabolites and pathways associated with NAFLD by including both genetic and non-genetic factors in statistical analyses. We genotyped six genetic variants in the *PNPLA3, TM6SF2, MBOAT7, GCKR*, *PPP1R3B*, and *HSD17B13* genes reported to be associated with NAFLD. Non-targeted metabolomic profiling was performed from plasma samples. We applied a previously validated fatty liver index to identify participants with NAFLD. First, we associated the six genetic variants with 1098 metabolites in 2 339 men without NAFLD to determine the effects of the genetic variants on metabolites, and then in 2 535 men with NAFLD to determine the joint effects of genetic variants and non-genetic factors on metabolites. We identified several novel metabolites and metabolic pathways, especially for *PNPLA3, GCKR,* and *PPP1R38* variants relevant to the pathophysiology of NAFLD. Importantly, we showed that each genetic variant for NAFLD had a specific metabolite signature. The plasma metabolite signature was unique for each genetic variant, suggesting that several metabolites and different pathways are involved in the risk of NAFLD. The FLI index reliably identifies metabolites for NAFLD in large population-based studies.

## 1. Introduction

Non-alcoholic fatty liver disease (NAFLD) is characterized by lipid deposition (>5% of liver weight) in the liver not related to alcohol consumption. NAFLD is the most common manifestation of chronic liver disease in Western countries and is predicted to become the most common cause of liver transplantation by 2030 [1]. Excess accumulation of triacylglycerols (TAGs) in the liver can proceed to non-alcoholic steatohepatitis (NASH) and even to hepatocellular carcinoma. NAFLD also affects extra-hepatic organs and metabolic pathways [2] and increases the risk of type 2 diabetes, cardiovascular disease, and chronic kidney disease [3].

Both genetic and non-genetic factors are important in the pathophysiology of NAFLD [4]. Several genetic variants have been reported to be associated with NAFLD in genome-wide association studies. A nonsynonymous rs738409-G variant in *PNPLA3* (patatin-like phospholipase domain-containing 3) encodes an amino acid substitution of I148M that is recognized as the most important genetic variant for the risk of NAFLD and NASH [5,6,7]. *PNPLA3* has TAG hydrolysis activity, and the accumulation of inactive *PNPLA3* leads to an increase in TAGs in the liver [6,8]. 

*TM6SF2* (transmembrane 6 superfamily member 2) encodes the *TM6SF2* protein and is involved in the assembly and lipidation of very-low-density lipoprotein (VLDL) particles [9,10]. *MBOAT7* (membrane-bound O-acyltransferase domain-containing 7 gene) encodes for the *MBOAT7* protein which is an enzyme involved in the remodeling of phospholipid acyl-chains and transfers polyunsaturated fatty acids (PUFAs) to lysophospholipids [10,11]. *GCKR* (glucokinase regulatory protein gene) encodes the glucokinase regulatory protein, which inhibits glucokinase in the liver [12]. 

Genetic variants in the *PPP1R3B* [13] and *HSD17B13* genes [14] are associated with a decreased risk of NAFLD. The *PPP1R3B* variant increases hepatic glycogen accumulation via activation of glycogen synthase and inactivation of glycogen phosphorylase [15]. The *HSD17B13* rs72613567:TA variant has an insertion of the A allele (TA/TA-homozygous) and encodes for the hepatic lipid droplet protein hydroxysteroid 17-beta dehydrogenase 13 and reduces the risk of liver fibrosis and steatosis [14,16].

Non-genetic factors are important in the pathogenesis of NAFLD. The increasing prevalence of NAFLD is closely related to the epidemic of obesity, as well as to insulin resistance and type 2 diabetes [17,18]. Therefore, metabolites associated with NAFLD reflect the effects of both genetic and non-genetic factors in the pathophysiology of this disease. However, only a few studies have tried to distinguish genetic and non-genetic components [19]. The gold standard methods for the diagnosis of NAFLD and NASH are liver biopsy and magnetic resonance imaging. These methods are expensive, and, therefore, the sample size is often limited in studies on NAFLD. Recently, a fatty liver index (FLI) based on the measurement of waist circumference, body mass index (BMI), TAG and gamma-glutamyl-transferase has been developed and validated as a non-invasive, practical, and efficient technique to diagnose NAFLD in large population studies [20]. The FLI can accurately identify NAFLD (high sensitivity and specificity) with an optimal cut-off point of 30 and suggests that participants with FLI ≥ 60 very likely have NAFLD, whereas participants with FLI < 30 likely do not have NAFLD [20].

We performed a comprehensive study to understand the effects of genetic variants in the metabolic pathways involved in the development of NAFLD (FLI ≥ 80) in men recruited from the METabolic Syndrome in Men (METSIM) study. Compared to the original publication [20], we applied an even more stringent criterion, 80, for the upper part of the FLI distribution, to be sure that these men had NAFLD. In statistical analyses, we first analyzed the effects of the six genetic variants separately on 1098 metabolites in 2 339 men without NAFLD (FLI < 30). This analysis reflects an isolated effect of each genetic variant on metabolite concentrations. We did not generate a polygenic risk score for NAFLD, because this gives only the joint effects of all genetic variants on metabolites and not a metabolite signature for each genetic variant. Our approach makes it possible to identify several independent metabolites and pathways leading to NAFLD. Finally, we analyzed the effects of these genetic variants with 1098 metabolites in 2 535 men with NAFLD. This analysis reflects the effects of genetic (genetic variants) and non-genetic effects (obesity, central obesity, insulin resistance, and type 2 diabetes) on metabolite concentrations. 

## 2. Materials and Methods

### 2.1. Study Population

The participants were recruited from the METSIM study, comprising 10,197 Finnish men randomly selected from the population register of Kuopio town, eastern Finland, aged from 45 to 73 years, and examined in 2005–2010. The study design has been described previously in detail [21,22]. The study was approved by the Ethics Committee of the University of Kuopio and Kuopio University Hospital. All study participants gave written informed consent. The study protocol follows the ethical guidelines of the Declaration of Helsinki, as reflected in a priori approval by the institution’s human research committee.

### 2.2. Clinical and Laboratory Measurements

Height was measured without shoes to the nearest 0.5 cm. Weight was measured in light clothing with a calibrated digital scale (Seca 877, Hamburg, Germany). BMI was calculated as weight (kg) divided by height (m) squared. We measured waist circumference at the midpoint between the lateral iliac crest and the lowest rib. Laboratory studies after 12 h fasting included the following measurements: plasma glucose and insulin, lipids, lipoproteins, and mass spectrometry metabolomics (Metabolon, Durham, NC, USA). An oral glucose tolerance test was performed (75 g of glucose) to evaluate glucose tolerance according to American Diabetes Association criteria [23]. Clinical and laboratory measurement methods have been previously described [22]. Briefly, plasma glucose was measured by enzymatic hexokinase photometric assay (Konelab Systems Reagents, Thermo Fischer Scientific, Vantaa, Finland). Insulin was determined by immunoassay (ADVIA Centaur Insulin IRI, no 02230141, Siemens Medical Solutions Diagnostics, Tarrytown, NY, USA). Total TAGs, plasma free fatty acids (FFAs), high-density lipoprotein cholesterol (HDLC), and low-density lipoprotein cholesterol (LDLC) were measured using enzymatic colorimetric tests (Konelab Systems Reagents; Thermo Fisher Scientific, Vantaa, Finland). Plasma adiponectin was measured using ELISA (human adiponectin ELISA kit; Linco Research, St. Charles, MI, USA), and serum alanine aminotransferase (ALT) and gamma-glutamyl transferase (GGT) by enzymatic photometric tests (Konelab Reagent System, Thermo Fisher Scientific, Vantaa, Finland). High-sensitivity C-reactive protein (hs-CRP) was assayed using kinetic immunoturbidimetry (near-infrared particle immunoassay; IMMAGE Immunochemistry System; Beckman Coulter, Fullerton, CA, USA). DNA was extracted from white blood cells.

### 2.3. Metabolomics Analysis

Non-targeted metabolomic profiling was performed at Metabolon, Inc. (Durham, NC, USA) on EDTA plasma samples obtained after an overnight fast. Briefly, methanol extraction of biochemicals was followed by non-targeted relative quantitative liquid chromatography–tandem mass spectrometry (LC-MS/MS). The Metabolon DiscoveryHD4 platform was applied to assay named metabolites. Samples were randomized across batches. Batches contained ~144 METSIM samples and 20 well-characterized human-EDTA plasma samples for quality control. All samples were processed together for peak quantification and data scaling. We quantified raw mass spectrometry peaks for each metabolite using the area under the curve and evaluated overall process variability using the median-relative standard deviation for endogenous metabolites present in all 20 technical replicates in each batch. We adjusted for variation caused by day-to-day instrument tuning differences and columns used for biochemical extraction by scaling the raw peak quantifications to the median for each metabolite by the Metabolon batch. We included in the statistical analysis 1098 metabolites, having data for at least 1000 men.

### 2.4. Calculations

The Matsuda Insulin Sensitivity Index (Matsuda ISI) was calculated as previously described [22,24]. The selection of Matsuda ISI as a marker of insulin sensitivity was based on our previous validation study (22). FLI was calculated using the following formula: FLI = (exp(0.953 × loge(TAG) + 0.139 × BMI + 0.718 × loge(GGT) + 0.053 × waist circumference –15.745))/(1 + exp(0.953 × loge(TAG) + 0.139 × BMI+ 0.718 × loge(GGT) + 0.053 × waist circumference–15.745)) × 100. FLI ≥ 80 indicates that the participant has NAFLD, and FLI < 30 indicates that the participants do not have NAFLD [20].

### 2.5. Genetic Analysis

We genotyped the genetic variants *PNPLA3* rs738409-G, *TM6SF2* rs58542926-T, *MBOAT7* rs641738-T, *GCKR* rs780094-T, *PPP1R3B* rs4841132-A, and *HSD17B13* rs72613567:TA using specific TaqMan assays (ThermoFisher) in a 7500 Real-Time PCR System (Applied Biosystems) or the Sequenom iPlex Gold SBE assay at the National Human Genome Research Institute at the National Institutes of Health as previously described [25]. 

### 2.6. Statistical Analysis

All statistical analyses were performed using IBM SPSS Statistics 27. All variables were log-transformed to correct for their skewed distribution. *p* < 4.5 × 10^−5^ was considered statistically significant given the 1098 metabolites measured and 6 genetic variants included in statistical analyses. We used ANOVA to calculate statistical differences in clinical and laboratory parameters between the men without (FLI < 30) and with NAFLD (FLI > 80). We applied linear regression analysis between the risk alleles of *PNPLA3* rs738409-G, *TM6SF2* rs58542926-T, *MBOAT7* rs641738-T, *GCKR* rs780094-T, *PPP1R3B* rs4841132-A, and *HSD17B13* rs72613567:TA and the metabolites (N = 1098) in men without and with NAFLD. The results are given as standardized beta coefficients, SE, and *p* values with each metabolite as a dependent variable. We used the Z-test to compare the statistical significance between two betas from linear regression [26].

## 3. Results

### 3.1. Clinical and Laboratory Measurements

Except for age, all other laboratory and clinical parameters showed highly statistically significant differences between men without NAFLD (FLI 18.9 ± 7.0) and with NAFLD (FLI 90.5 ± 5.8) (Table 1). Men with NAFLD were more obese and more centrally obese, had higher concentrations of ALT, LDLC, TAG, glucose, insulin, hs-CRP, and FFAs, and more often had type 2 diabetes than men without NAFLD.

We associated six genetic variants associated with NAFLD with clinical and laboratory measurements (Supplementary Table S1). Only a few statistically significant associations were found in men without NAFLD. The *PNPLA3* variant was significantly associated with decreased fasting insulin, the *GCKR* variant with increased TAGs, and the *PPP1R3B* variant with decreased HDLC and fasting FFAs. In men with NAFLD, the *PNPLA3* variant was associated with increased ALT, the *TM6SF2* variant with decreased TAGs, the *GCKR* variant with increased TAGs, the *PPP1R3B* variant with decreased HDLC and decreased FFAs, and the *HSD17B13* variant with decreased ALT and TAGs. None of these genetic variants were associated with insulin Matsuda ISI, obesity, or glucose or insulin concentrations.

### 3.2. Association of the Six NAFDL Genetic Risk Variants with Metabolites

**Figure 1:**
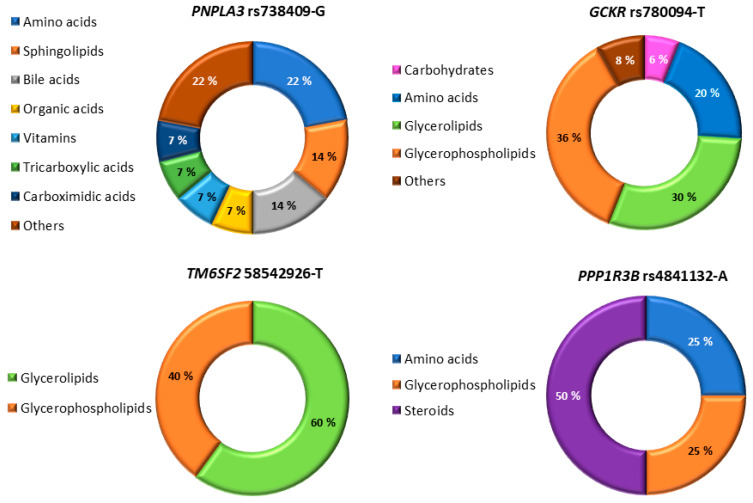
Metabolite subgroups associated with four genetic variants in men with NAFLD.

First, we investigated the effects of the six genetic variants (*PNPLA3* rs738409-G, *TM6SF2* rs58542926-T, *MBOAT7* rs641738-T, *GCKR* rs780094-T, *PPP1R3B* rs4841132-A, *HSD17B13* rs72613567:TA) on metabolites in men without NAFLD (Table 2). This analysis reflects an isolated effect of each genetic variant on metabolite concentrations. Next, we investigated the effects of the genetic variants on metabolites in men with NAFLD (Supplementary Table S1). This analysis reflects the combined effects of the genetic variants and non-genetic risk factors for NAFLD (obesity, type 2 diabetes, insulin resistance) on metabolite concentrations. If the effects of the genetic variants on metabolite concentrations are not significantly different between men with and without NAFLD, then the effects on metabolites are explained largely by genetic variants. Figure 1 shows the groups of metabolites associated with NAFLD. The *PNPLA3* variant was associated with several metabolite groups, whereas the *TM6SF2* and *GCKR* variants were mainly associated with glycerolipids and glycerophospholipids and the *PPP1R3B* variant with steroids, amino acids, and glycerophospholipids.

*PNPLA3* rs738409-G. In men without NAFLD, we found only one significant association of the *PNPLA3* variant with nicotinate ribonucleoside (organo-oxygen compound, beta −0.093, *p* = 3.2 × 10^−5^) (Table 2). In men with NAFLD, we found 12 novel associations of the *PNPLA3* variant with metabolites (Supplementary Table S2). The strongest association of the *PNPLA3* variant was with 3-ureidopropionate (beta 0.136, *p* = 1.8 × 10^-10^). Other significant novel associations were with two amino acids (serine, N-acetylisoputreanine), two sphingolipids, one fatty acid, two bile acids (taurochenodeoxycolate, glycochenodeoxycholate), one tricarboxylacid (aconitate), and one carboxymidic acid (N1/N8 acetylspermide). All metabolites except for ceramide (d16:1/24:1; d18:1/22:1) were positively associated with the *PNPLA3* variant. 

*TM6SF2* rs58542926-T. We found one significant association of the *TM6SF2* variant with oleoyl-arachidonoyl-glycerol (18:1/20:4) [2] (beta −0.107, *p*= 3.7 × 10^−6^) in men without NAFLD (Table 2) and novel statistically significant inverse associations with three glycerolipids and two glycerophospholipids in men with NAFLD (Supplementary Table S2). 

*MBOAT7* rs641738-T. In men without NAFLD, we found positive associations of the *MBOAT7* variant with seven glycerophospholipids (four phosphatidylinositols (PIs) and three lyso-phosphatidylinositols (lyso-PIs)) and three inverse associations with two PIs and one lyso-PI, all containing arachidonic acid in their acyl chain (Table 2). In men with NAFLD, we found statistically significant associations with the same metabolites as in men without NAFLD (Supplementary Table S2). Beta coefficients were not significantly different between men with and without NAFLD, which suggests that we did not find any specific metabolites associated with NAFLD. 

*GCKR* rs780094. In men without NAFLD, we found a novel statistically significant inverse association of the *GCKR* variant with mannonate (beta 0.181, *p* = 1.0 × 10^−17^), and a previously reported association with mannose (Table 2). In men with NAFLD, we found novel statistically significant associations of the *GCKR* variant with 28 metabolites including amino acids (four metabolites in branched-chain amino acid pathways), lipid pathways (five glycerolipids, ten glycerophospholipids), and α-ketobutyrate (Supplementary Table S2). We found the strongest novel positive associations of the *GCKR* variant with 1-carboxyethylleucine (beta 0.133, *p* = 2.2 × 10^−9^) and 1-carboxyethylvaline (beta 0.125, *p* = 5.7 × 10^−9^). We also found novel statistically significant inverse associations with the ɣ-glutamyl amino acids gamma-glutamylthreonine and gamma-glutamyl-citrulline.

*PPP1R3B* rs4841132-A. In men without NAFLD, we found novel statistically significant inverse associations of the *PPP1R3B* variant with hexanoylglutamine, 3-hydroxybutyrate, retinol, and hydroxypalmitoyl sphingomyelin, and confirmed one previously reported positive association of the *PPP1R3B* variant with glycine (Table 2). The strongest novel association of the *PPP1R3B* variant was found with 3-hydroxybutyrate (beta −0.115, *p* = 6.1 × 10^−8^). In men with NAFLD, we found novel statistically significant positive associations of the *PPP1R3B* variant with nine metabolites, including two lyso-phosphatidylethanolamines (lyso-PEs) and four steroids, and novel statistically significant inverse associations with two metabolites belonging to the tryptophan pathway, N-acetylkynurenine [2] (beta −0.098, *p* = 2.4^−6^) and xanthurenate (beta −0.085, *p* = 3.6 × 10^−5^) (Supplementary Table S2).

*HSD17B13* rs72613567. In men without NAFLD, we did not find any statistically significant association of the *HSD17B13* variant with metabolites (Table 2). In men with NAFLD, we found novel statistically significant positive associations with two glycerophospholipids, 1-stearoyl-2-linoleoyl-GPE (18:0/18:2) and 1-stearoyl-2-oleoyl-GPE (18:0/18:1) (Supplementary Table S2).

## 4. Discussion

Our study is the first large population-based study investigating the association of six genetic variants increasing (*PNPLA3*, *TM6SF2*, *GCKR*, *MBOAT7*) and two genetic variants decreasing (*PPP1R3B*, HDS17B13) the risk of NAFLD. We identified several novel variant–metabolite associations in different metabolic pathways relevant to the pathophysiology of NAFLD. Importantly, we showed that each genetic variant for NAFLD has a specific metabolite signature.

NAFLD is explained by both genetic and non-genetic factors. Among non-genetic factors, obesity, insulin resistance, and type 2 diabetes play important roles in the risk of NAFLD [19]. None of the six genetic variants for NAFLD were associated with obesity, hyperglycemia, or insulin resistance in our study. This implies that obesity, insulin resistance, and hyperglycemia are regulated by metabolic changes independently of genetic factors.

*PNPLA3* rs738409-G. The *PNPLA3* variant is the most important genetic variant associated with NAFLD. A recent study has shown that the steatosis associated with the *PNPLA3* variant is caused by the accumulation of *PNPLA3* protein on lipid droplets [27]. In men with NAFLD, we found 12 novel associations of the *PNPLA3* variant with metabolites belonging to different metabolic pathways. We also found decreased levels of N-acetylmethionine (NAM) in carriers of the *PNPLA3* variant having NAFLD, similarly as has been published for patients with NASH [28].

**Figure 2:**
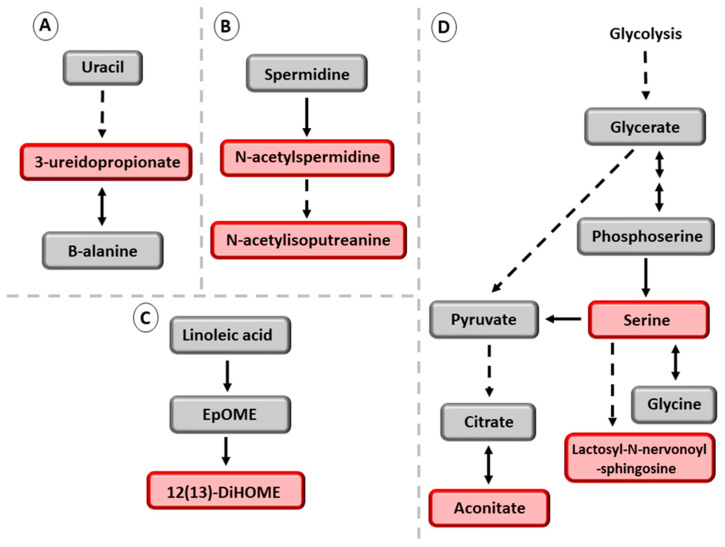
Novel metabolites associated with *PNPLA3* rs738409-G in men with NAFLD. Red color indicates increased concentration of the metabolites. Grey color indicates that these metabolites were not significantly associated with *PNPLA3* rs738409-G. (**A**) metabolites belonging to the uracil pathway. (**B**) downstream metabolites belonging to the spermidine pathway. (**C**) downstream metabolites of the linoleic acid pathway. (**D**) metabolites coming from glycolysis pathway. Abbreviations: EpOME, epoxyoctadecenoic acid; 12(13)DiHOME, 12,13-dihydroxy-9-octadecenoic acid.

The most significant positive association of the *PNPLA3* variant was with 3-ureidopropionate (3-UPA), a metabolite in pyrimidine degradation (Figure 2A). 3-UPA has not previously been linked to NAFLD in human studies, but, in mice, liver pyrimidine degradation was associated with lipid accumulation in the liver [29]. The β-ureidopropionase enzyme catalyzes the conversion of 3-UPA to β-alanine [30,31,32]. High concentrations of 3-UPA increase reactive oxygen species (ROS) production and inhibit mitochondrial energy metabolism in complex V (ATP synthase) [33]. Decreased activity of mitochondrial respiratory complexes I, III, IV and V has been previously reported in NASH [34,35]. Our novel findings were also that the *PNPLA3* variant had significant positive associations with downstream metabolites of spermidine catabolism, N-acetylspermidine, and N-acetylisoputreanine in men with NAFLD (Figure 2B). We also found in men with NAFLD increased levels of 12,13-diHOME (Figure 2C), tricarboxylic acid aconitinate, a metabolite involved in the mitochondrial tricarboxylic acid (TCA) cycle, and carboximidic acid N1/N8-acetylspermidine (Figure 2D), as well as elevated concentrations of two bile acids, taurochenodeoxycholate and glycohenodeoxycholate. A previous study has reported a significant increase in the fasting concentration of taurochenodeoxycholate in patients with NASH [36,37].

*TM6SF2* rs58542926-T. We found statistically significant novel inverse associations of the *TM6SF2* variant with three diacylglycerols (DAGs) and two phosphatidylcholines (PCs) in men with NAFLD. These lipids have PUFAs in their acyl chain. Our findings agree with a previous study showing that the *TM6SF2* variant contributes to steatosis by increasing the synthesis of PUFAs, whereas the synthesis of complex lipids containing PUFAs is impaired [38]. We also confirmed that the *TM6SF2* variant was significantly and inversely associated with TAG concentrations [39]. Our results support the notion that the *TM6SF2* variant is involved in the development of NAFLD by affecting the fatty acyl chain composition of PCs, impairing VLDL assembling and secretion, and consequently promoting lipid accumulation and inflammation in the liver [38].

*MBOAT7* rs641738C>T. *MBOAT7* participates in acyl chain remodeling of PIs [40]. We found novel statistically significant positive associations with four PIs and three lyso-PIs and novel inverse associations with two PIs and one lyso-PI in men both with and without NAFLD, suggesting that these associations were largely explained by the *MBOAT7* variant. All of these lipids contained arachidonic acid in their acyl chain and were inversely associated with the *MBOAT7* variant. Elevated concentrations of circulating proinflammatory metabolites of arachidonic acid and lyso-PIs have been reported in patients with NASH [41,42]. Moreover, lyso-PIs activate stellate cell compartments and trigger fibrosis in the liver [43].

*GCKR* rs780094-T. The *GCKR* variant was significantly and inversely associated with mannose in men with and without NAFLD, suggesting that this association is explained largely by genetic factors. Mannose is an indicator of hepatic glycogen breakdown, and it may reflect hepatic GCK and *GCKR* activity [44,45]. We found 28 novel significant associations of the *GCKR* variant with metabolites (mainly glycerolipids, glycerophospholipids, and amino acids) in men with NAFLD, and replicated previously reported associations with pyruvate, lactate, threonine, 3-aminoisobutyrate, glycerolipids, and glycerophospholipids [46]. Importantly, many of the glycerolipids and glycerophospholipids have palmitic acid in their side chain, supporting the role of the *GCKR* variant in the pathogenesis of NAFLD via generation of malonyl-CoA and an increase in de novo lipogenesis [47]. In addition, a recent study demonstrated that a high hepatic cytosolic NADH/NAD+ ratio stimulates fatty acid synthesis resulting in accumulation of TAGs in the liver; inhibits gluconeogenesis by preventing the oxidation of lactate to pyruvate [48]; and increases the generation of ROS due to the leak-out of electrons from mitochondrial complexes [49].

**Figure 3:**
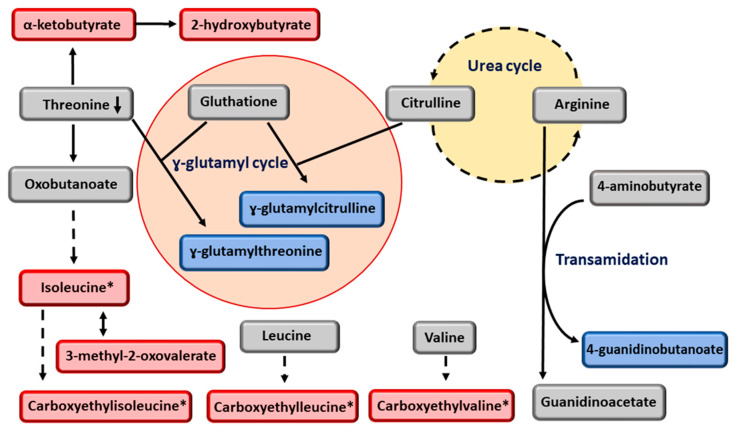
Novel metabolites associated with *GCKR* rs780094-T in men with NAFLD. Red color indicates increased and blue color decreased concentration of the metabolites. Grey color indicates that these metabolites were not significantly associated with *GCKR* rs780094-T. * Metabolites from gut microbiota.

We found novel positive associations of the *GCKR* variant with branched-chain amino acids (leucine, isoleucine, and valine) and their downstream metabolites (3-methyl-2-oxovaleriate, carboxyethyl-leucine, carboxyethyl-isoleucine, and carboxyethyl-valine) (Figure 3). Branched-chain amino acid concentrations in the liver have been reported to be increased in NASH compared to NAFLD [50]. We confirmed an inverse association of the *GCKR* variant with threonine as previously published [47]. Isoleucine, α-ketobutyrate, and ɣ-glutamyl-threonine are downstream metabolites of threonine [51,52]. Citrulline, a metabolite originating from the urea cycle, is also involved in the ɣ-glutamyl cycle [51], producing ɣ-glutamyl citrulline. The *GCKR* variant was significantly and inversely associated with citrulline in men with NAFLD. We also found an inverse association of the *GCKR* variant with 4-guanidinobutanoate, a downstream metabolite of arginine. The inverse associations of the *GCKR* variant with ɣ-glutamyl citrulline and 4-guanidinobutanoate might reflect poor regulation of the urea cycle in men with NAFLD.

**Figure 4:**
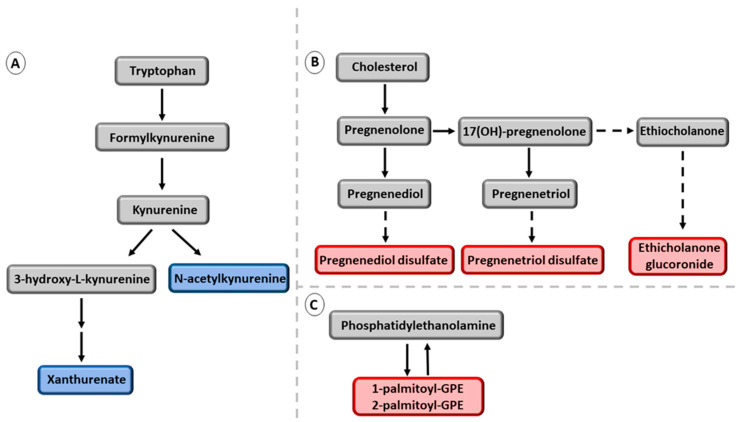
Novel metabolites associated with *PPP1R3B* rs4841132-A in men with NAFLD. (**A**) downstream metabolites of the tryptophan pathway. (**B**) metabolites belonging to the pregnenolone pathway. (**C**) metabolites belonging to the phosphatidylethanolamide pathway. Red color indicates increased and blue color decreased concentration of the metabolites. Grey color indicates that these metabolites were not significantly associated with *PPP1R3B* rs4841132-A.

*PPP1R3B* rs4841132-A. The *PPP1R3B* variant has been associated with an increase in liver glycogen content [13] and a decreased risk of liver steatosis, fibrosis, and HCC [53]. We report for the first time a detailed metabolic signature of the *PPP1R3B* variant (Figure 4). We found a novel inverse association of this variant with hexanoylglutamine and a previously reported positive association with glycine [54] in men both with and without NAFLD, suggesting that the *PPP1R3B* variant is largely responsible for these associations. In our study, the *PPP1R3B* variant was associated with decreased concentrations of fasting FFAs. In men with NAFLD, we found novel inverse associations of the *PPP1R3B* variant with downstream metabolites of tryptophan, N-acetylkynurenine, and xanthurenate, suggesting a decrease in inflammatory reactions [55]. We also found novel positive associations with three steroids, but the clinical significance of this finding remains to be determined.

*HSD17B13*. We found that the *HSD17B13* variant was significantly associated with decreased ALT levels, in agreement with a previous study [16]. The *HSD17B13* variant was also significantly associated with two glycerophospholipids in men with NAFLD. Our results agree with a previous study demonstrating that phospholipids were enriched in the liver in carriers of the *HSD17B13* variant [56].

**Figure 5:**
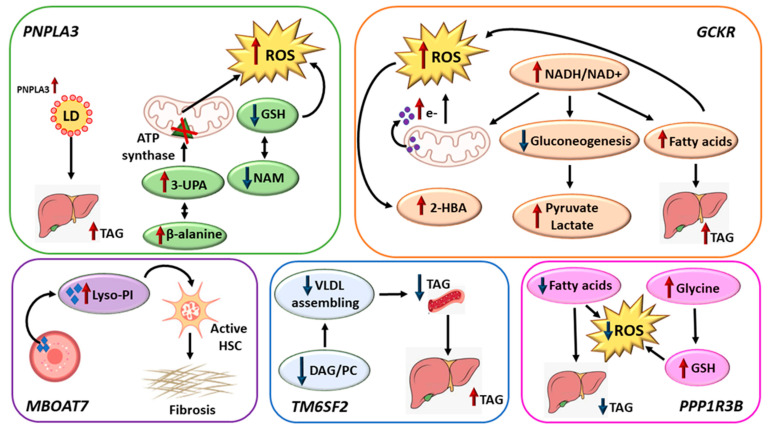
Summary of the main pathways of the five genetic variants associated with NAFLD. Abbreviations: 2-HBA, 2-hydroxybutyrate; 3-UPA, 3-ureidopropionate; ATP, adenosine triphosphate; DAG, diacylglycerol; e-, electrons; GSH, glutathione; HSC, hepatic stellate cells; LD, lipid droplet; Lyso-PI, lyso-phosphatidylinositol; NAD+, nicotinamide adenine dinucleotide; NADH, reduced nicotinamide adenine dinucleotide; NAM, N-acetylmethionine; PC, phosphatidylcholine; ROS, reactive oxygen species; TAG, triacylglycerol; VLDL, very-low-density lipoprotein.

In summary, we identified several novel metabolites for NAFLD, especially associated with the *PNPLA3*, *GCKR*, and *PPP1R3B* variants (Figure 5). Importantly, each genetic variant had its own metabolite signature, suggesting that multiple metabolic pathways contribute to NAFLD. The *PNPLA3* variant leads to accumulation of the *PNPLA3* protein on the surface of lipid droplets, causing fat accumulation in the liver. Our novel finding was an increased concentration of 3-UPA in men with NAFLD, which inhibits ATP synthase in mitochondria resulting in the generation of ROS. The *GCKR* variant increases the NADH/NAD+ ratio, which leads to leaking of electrons from the mitochondria, increasing the generation of ROS, a decrease in gluconeogenesis, and an increase in FA synthesis triggering the generation of ROS and increased TAG levels in the liver. The *PPP1R3B* variant increases the levels of glycine, a substrate for glutathione (GSH), counteracting ROS generation. *MBOAT7* variant increases concentrations of lyso-PI activating hepatic stellate cells triggering fibrosis in the liver. The *TM6SF2* variant decreases the assembly of VLDL particles and their export from the liver, leading to low TAG concentrations in the bloodstream and accumulation of TAG in the liver.

The strength of our study is the large size of the METSIM study, allowing us to find novel metabolites associated with genetic variants for NAFLD. In metabolite analyses, we applied a very conservative *p* value threshold. The limitations of our study are that it was cross-sectional, and only middle-aged and elderly Finnish men were included in the study. Therefore, we do not know if the results are valid for women, all age groups, or other ethnic and racial groups. We did not directly measure liver fat content in men to confirm a clinical diagnosis of NAFLD. Instead, we applied the FLI index that has been previously validated [20]. In our study, we used an even more stringent criterion, 80, for the upper part of the FLI distribution, compared to 60 in the original publication [20]. We also replicated several previously published metabolites for NAFLD, which provides evidence that the FLI index is a reliable marker for NAFLD in large population-based studies.

In conclusion, we identified several novel metabolites associated with genetic variants for NAFDL. The plasma metabolite signature was unique for each variant, suggesting that several different metabolites and pathways are involved in the pathophysiology of NAFLD. Our study also suggests that the FLI index reliably identifies metabolites for NAFDL in large population-based studies.

## Figures and Tables

**Table 1:** Baseline characteristics of men included in the study.

	Men without NAFLD (*n* = 2339)		Men with NAFLD (*n* = 2535)		
	Mean or %	SD	Mean or %	SD	*p* Value
Age, years	57.47	7.29	57.24	6.9	0.267
Body mass index, kg/m^2^	23.24	1.81	32.18	4.20	<0.001
Waist circumference, cm	86.79	5.38	112.28	10.48	<0.001
ALT, U/L	22.90	10.26	45.09	27.20	<0.001
LDL cholesterol, mmol/L	3.12	0.79	3.36	0.98	<0.001
HDL cholesterol, mmol/L	1.65	0.41	1.25	0.33	<0.001
Total triglycerides, mmol/L	0.89	0.30	2.24	1.54	<0.001
Matsuda ISI	10.33	4.59	3.40	2.03	<0.001
Plasma adiponectin, ug/mL	9.22	5.07	6.82	3.61	<0.001
hs-CRP, mg/l	1.34	3.24	3.44	6.46	<0.001
Fasting plasma glucose, mmol/L	5.60	0.59	6.48	1.57	<0.001
2-h plasma glucose, mmol/L	5.53	1.64	7.80	3.04	<0.001
Fasting plasma insulin, mU/L	4.83	2.46	17.72	20.85	<0.001
2-h plasma insulin, mU/L	27.93	24.68	96.21	81.56	<0.001
Fasting plasma FFA, mmol/L	0.35	0.15	0.43	0.17	<0.001
Type 2 diabetes, %	4.20	-	29.8	-	<0.001

**Table 2:** Associations of *PNPLA3* rs738409-G, *TM6SF2* rs58542926-T, *MBOAT7* rs641738-T, *GCKR* rs780094-T and *PPP1R3B* rs4841132-A with metabolites in men without NAFLD.

*PNPLA3* rs738409-G	Direct Parent	Beta	SE	*p* Value	Novel
**Organooxygen compound**					
Nicotinate ribonucleoside	Glycosylamine	−0.093	0.037	3.2 × 10^−5^	Yes
***TM6SF2*** **rs58542926-T**					
**Glycerolipid**					
Oleoyl-arachidonoyl-glycerol (18:1/20:4 [2])	Diacylglycerol	−0.107	0.062	3.7 × 10^−6^	Yes
***MBOAT7*** **rs641738-T**					
**Glycerophospholipid**					
1-stearoyl-2-arachidonoyl-GPI (18:0/20:4)	PI	−0.207	0.029	<1 × 10^−50^	Yes
1-palmitoyl-2-linoleoyl-GPI (16:0/18:2)	PI	0.177	0.029	3.1 × 10^−16^	Yes
1-palmitoyl-2-oleoyl-GPI (16:0/18:1)	PI	0.173	0.028	1.2 × 10^−15^	Yes
1-stearoyl-2-linoleoyl-GPI (18:0/18:2)	PI	0.170	0.030	1.6 × 10^−15^	Yes
1-palmitoyl-2-arachidonoyl-GPI (16:0/20:4)	PI	−0.133	0.028	1.8x10^−9^	Yes
1-stearoyl-2-oleoyl-GPI (18:0/18:1)	PI	0.101	0.037	7.9 × 10^−05^	Yes
1-linoleoyl-GPI (18:2)	Lyso-PI	0.183	0.030	1.0 × 10^−17^	Yes
1-palmitoleoyl-GPI (16:1)	Lyso-PI	0.170	0.035	8.6 × 10^−11^	Yes
1-arachidonoyl-GPI (20:4)	Lyso-PI	−0.120	0.030	2.4 × 10^−8^	Yes
1-oleoyl-GPI (18:1)	Lyso-PI	0.114	0.030	1.0 × 10^−7^	Yes
***GCKR*** **rs780094-T**					
**Carbohydrate**					
Mannose	-	−0.277	0.027	<1 × 10^−50^	No
**Other metabolites**					
Mannonate	-	−0.181	0.027	1.0 × 10^−17^	Yes
***PPP1R3B*** **rs4841132-A**					
**Amino acids**					
Glycine	-	0.138	0.039	9.7 × 10^−11^	No
Hexanoylglutamine	-	−0.114	0.043	1.6 × 10^−7^	Yes
**Hydroxy acid**					
3-hydroxybutyrate (BHBA)	β-hydroxy acid	−0.115	0.043	6.1 × 10^−8^	Yes
**Other metabolites**					
Retinol (Vitamin A)	Vitamin	−0.106	0.036	6.9 × 10^−7^	Yes
Hydroxypalmitoyl sphingomyelin (d18:1/16:0 (OH))	-	−0.099	0.036	3.9 × 10^−6^	Yes
Glutamine conjugate of C6H10O22	-	−0.089	0.044	3.7 × 10^−5^	Yes

Abbreviations: Lyso-PI, lyso-phosphatidylinositol; PI, phosphatidylinositol. *HSD17B13* rs72613567:TA did not show any significant results.

## Data Availability

All datasets generated during the current study can be found within the manuscript. Other datasets generated during and/or analyzed during the current study are available from the corresponding author on reasonable request.

## Supplementary Materials

The following supporting information can be downloaded at: https://www.mdpi.com/article/10.3390/metabo13020267/s1, Table S1: Associations of PNPLA3 rs738409-G, TM6SF2 rs58542926-T, MBOAT7 rs641738-T, GCKR rs780094-T, PPP1R3B rs4841132-A and HSD17B13 rs72613567:TA with laboratory parameters, Matsuda ISI and body mass index; Table S2: Associations of PNPLA3 rs738409-G, TM6SF2 rs58542926-T, GCKR rs780094-T, PPP1R3B rs4841132-A and HSD17B13 rs72613567:TA with metabolites in participants with NAFLD.
