# Supplementary material for: Plasma Metabolite Signatures in Male Carriers of Genetic Variants Associated with Non-Alcoholic Fatty Liver Disease
Source: Metabolites. 2023 Feb 13;13(2):267. doi: 10.3390/metabo13020267 (PMC9964056; doi:10.3390/metabo13020267)
Supplement: Supplementary file 1 [file metabolites-13-00267-s001.zip › metabolites-2201162-supplementary.pdf]

Supplementary Table S1. Associations of *PNPLA3* rs738409-G, *TM6SF2* rs58542926-T, *MBOAT7* rs641738-T, *GCKR* rs780094-T, *PPP1R3B* rs4841132-A and *HSD17B13* rs72613567:TA with laboratory parameters, Matsuda ISI and body mass index

	**Participants without NAFLD**			**Participants with NAFLD**		
***PNPLA3* rs738409-G**	**Beta**	**SE**	**P value**	**Beta**	**SE**	**P value**
ALT (U/l)	-0,007	0,070	0,747	0,318	0,051	**6,6E-10**
LDLC (mmol/l)	0,015	0,107	0,487	0,013	0,088	0,887
HDLC (mmol/l)	-0,011	0,118	0,611	0,024	0,106	0,822
TAG (mmol/l)	-0,001	0,090	0,968	-0,153	0,056	0,006
BMI (kg/m2)	0,020	0,363	0,344	-0,268	0,220	0,222
Matsuda ISI	0,057	0,065	0,008	-0,120	0,053	0,023
Adiponectin (ug/ml)	-0,002	0,064	0,939	0,073	0,060	0,224
hs-CRP (mg/l)	0,046	0,028	0,030	-0,019	0,028	0,498
Fasting glucose (mmol/l)	-0,030	0,294	0,158	0,214	0,142	0,133
2h glucose (mmol/l)	-0,038	0,104	0,076	0,046	0,084	0,581
Fasting insulin	-0,066	0,068	**0,002**	0,082	0,044	0,061
2h insulin (mU/l)	-0,042	0,039	0,051	0,061	0,036	0,086
Fasting FFA (mmol/l)	-0,034	0,067	0,106	-0,098	0,070	0,161
***TM6SF2* rs58542926-T**	**Beta**	**SE**	**P value**	**Beta**	**SE**	**P value**
ALT (U/l)	-0,002	0,039	0,938	0,029	0,030	0,179
LDLC (mmol/l)	-0,030	0,060	0,146	-0,033	0,052	0,125
HDLC (mmol/l)	-0,008	0,066	0,690	0,017	0,062	0,435
TAG (mmol/l)	-0,059	0,050	0,005	-0,078	0,033	**<0.001**
BMI (kg/m2)	0,019	0,203	0,376	-0,006	0,129	0,792
Matsuda ISI (mg/dl, mU/l)	0,004	0,036	0,847	-0,010	0,031	0,690
Adiponectin (ug/ml)	-0,025	0,036	0,239	0,018	0,035	0,415
hs-CRP (mg/l)	-0,016	0,016	0,438	-0,007	0,016	0,751
Fasting glucose (mmol/l)	-0,002	0,164	0,924	0,005	0,080	0,827
2h glucose (mmol/l)	0,011	0,058	0,597	-0,024	0,049	0,321
Fasting insulin	-0,004	0,038	0,831	0,027	0,025	0,213
2h insulin (mU/l)	0,002	0,022	0,914	0,004	0,021	0,869
Fasting FFA (mmol/l)	-0,024	0,037	0,251	-0,042	0,041	0,052
***MBOAT7* rs641738-T**	**Beta**	**SE**	**P value**	**Beta**	**SE**	**P value**
ALT (U/l)	-0,0003	0,081	0,988	0,031	0,060	0,124
LDLC (mmol/l)	-0,017	0,124	0,414	0,003	0,103	0,873
HDLC (mmol/l)	-0,007	0,137	0,728	-0,015	0,123	0,444
TAG (mmol/l)	-0,005	0,105	0,801	-0,023	0,065	0,257
BMI (kg/m2)	0,013	0,423	0,521	-0,004	0,256	0,848
Matsuda ISI (mg/dl, mU/l)	0,021	0,075	0,318	-0,019	0,061	0,376

Adiponectin (ug/ml)	0,046	0,074	0,030	-0,051	0,070	0,010
hs-CRP (mg/l)	0,006	0,033	0,768	0,002	0,033	0,911
Fasting glucose (mmol/l)	-0,034	0,343	0,108	0,013	0,166	0,525
2-hour glucose (mmol/l)	0,019	0,122	0,361	0,005	0,097	0,814
Fasting insulin	-0,046	0,079	0,027	0,011	0,051	0,587
2-hour insulin (mU/l)	0,002	0,046	0,942	0,024	0,041	0,268
Fasting FFA (mmol/l)	0,034	0,078	0,101	-0,007	0,081	0,739
***GCKR* rs780094-T**	**Beta**	**SE**	**P value**	**Beta**	**SE**	**P value**
ALT (U/l)	-0,017	0,080	0,425	-0,001	0,061	0,962
LDLC (mmol/l)	0,021	0,123	0,320	0,029	0,104	0,149
HDLC (mmol/l)	0,004	0,135	0,835	-0,051	0,125	0,010
TAG (mmol/l)	0,066	0,103	**0,001**	0,131	0,066	**4,31E-11**
BMI (kg/m2)	0,008	0,416	0,689	-0,038	0,260	0,060
Matsuda ISI (mg/dl, mU/l)	0,039	0,074	0,065	0,053	0,063	0,014
Adiponectin (ug/ml)	0,009	0,073	0,674	-0,027	0,071	0,180
hs-CRP (mg/l)	-0,005	0,032	0,821	0,029	0,033	0,145
Fasting glucose (mmol/l)	-0,034	0,338	0,101	-0,044	0,168	0,026
2-hour glucose (mmol/l)	0,005	0,120	0,813	0,057	0,099	0,008
Fasting insulin	-0,038	0,078	0,066	-0,055	0,052	0,006
2-hour insulin (mU/l)	-0,023	0,045	0,284	-0,021	0,042	0,332
Fasting FFA (mmol/l)	-0,012	0,077	0,581	0,052	0,082	0,010
***PPP1R3B* rs4841132-A**	**Beta**	**SE**	**P value**	**Beta**	**SE**	**P value**
ALT (U/l)	-0,028	0,062	0,178	0,007	0,046	0,731
LDLC (mmol/l)	-0,018	0,095	0,400	-0,029	0,079	0,142
HDLC (mmol/l)	-0,085	0,105	**4,54E-05**	-0,064	0,095	**0,001**
TAG (mmol/l)	0,059	0,080	0,005	0,013	0,050	0,520
BMI (kg/m2)	-0,022	0,323	0,297	-0,016	0,197	0,428
Matsuda ISI (mg/dl, mU/l)	-0,011	0,058	0,611	0,000	0,047	0,993
Adiponectin (ug/ml)	-0,013	0,057	0,528	0,021	0,054	0,286
hs-CRP (mg/l)	-0,031	0,025	0,139	-0,042	0,025	0,037
Fasting glucose (mmol/l)	0,034	0,262	0,098	0,015	0,128	0,439
2-hour glucose (mmol/l)	-0,027	0,094	0,200	-0,054	0,074	0,012
Fasting insulin	0,033	0,061	0,111	0,020	0,039	0,309
2-hour insulin (mU/l)	-0,024	0,035	0,247	-0,042	0,032	0,051
Fasting FFA (mmol/l)	-0,083	0,060	**7,10E-05**	-0,078	0,062	**9,74E-05**
***HSD17B13* rs72613567:TA**	**Beta**	**SE**	**P value**	**Beta**	**SE**	**P value**
ALT (U/l)	-0,027	0,067	0,192	-0,074	0,050	**2,22E-04**
LDLC (mmol/l)	0,004	0,102	0,864	0,012	0,086	0,537

HDLC (mmol/l)	-0,012	0,113	0,566	-0,008	0,103	0,685
TAG (mmol/l)	0,023	0,086	0,282	0,066	0,055	**0,001**
BMI (kg/m2)	0,014	0,348	0,502	-0,027	0,215	0,182
Matsuda ISI (mg/dl, mU/l)	-0,022	0,062	0,296	0,006	0,052	0,794
Adiponectin (ug/ml)	-0,012	0,061	0,559	-0,010	0,058	0,614
hs-CRP (mg/l)	0,025	0,027	0,232	-0,010	0,027	0,613
Fasting glucose (mmol/l)	-0,008	0,282	0,702	-0,021	0,139	0,292
2-hour glucose (mmol/l)	0,014	0,100	0,511	-0,024	0,082	0,267
Fasting insulin	0,015	0,065	0,482	-0,016	0,043	0,429
2-hour insulin (mU/l)	0,012	0,038	0,561	-0,013	0,035	0,566
Fasting FFA (mmol/l)	0,015	0,064	0,479	-0,018	0,068	0,377

Abbreviations: ALT, alanine aminotransferase; LDLC, low density lipoprotein cholesterol; HDLC, high density lipoprotein cholesterol; TAG, triacylglycerol; BMI, body mass index; hs-CRP, high sensitivity C-reactive protein; FFA, free fatty acids. Results obtained using linear regression. P-values <0,0025 are statistically significant (14 varibles+6 genetic variants). Statistically significant results are marked in bold.

Supplementary Table S2. Associations of *PNPLA3* rs738409-G, *TM6SF2* rs58542926-T, *GCKR* rs780094-T, *PPP1R3B* rs4841132-A and *HSD17B13* rs72613567:TA with metabolites in participants with NAFLD

PNPLA3 rs738409-G	**Direct parent**	**Beta**	**SE**	**P-value**	**Novel**
**Organic acids**					
3-ureidopropionate	Urea	0,136	0,037	1,85E-10	Yes
**Amino acids**					
Serine	**-**	0,095	0,035	3,92E-06	Yes
N-acetylmethionine	Methionine derivative	-0,092	0,042	6,66E-06	No
N-acetylisoputreanine	ɣ-amino acid	0,107	0,036	1,70E-07	Yes
**Lipids**					
**Sphingolipids**					
Lactosyl-N-nervonoyl-sphingosine (d18:1/24:1)	Lactosyl ceramide	0,109	0,040	1,20E-06	Yes
Ceramide (d16:1/24:1; d18:1/22:1)	Ceramide	-0,099	0,037	1,70E-05	Yes
**Fatty acids**					
12-13DiHOME	Long chain fatty acid	0,114	0,040	2,95E-06	Yes
**Bile acids**					
Taurochenodeoxycholate	-	0,100	0,036	1,54E-06	Yes
Glycochenodeoxycholate	-	0,093	0,035	5,98E-06	Yes
**Other metabolites**					
Retinol (Vitamin A)	Vitamin	-0,090	0,037	1,27E-05	No
Glutamine conjugate of C6H10O21	-	0,090	0,032	1,40E-05	Yes
Branched-chain/straight-chain or cyclopropyl 10:1 fatty acid [1]	-	0,092	0,037	7,29E-06	Yes
Aconitate (cis or trans)	Tricarboxylic acids	0,085	0,035	3,27E-05	Yes
N1/N8 acetylspermidine	Carboxymidic acid	0,091	0,036	9,69E-06	Yes
TM6SF2					
**Lipids**					
**Glycerolipids**					

Palmitoleoyl-linoleoyl-glycerol (16:1/18:2)[1]	Diacylglycerol	-0,104	0,070	8,58E-06	Yes
Oleoyl-linoleoyl-glycerol (18:1/18:2) [1]	Diacylglycerol	-0,087	0,064	2,63E-05	Yes
Oleoyl-linoleoyl-glycerol (18:1/18:2 [2]	Diacylglycerol	-0,085	0,064	4,05E-05	Yes
**Glycerophospholipids**					
1-myristoyl-2-docosahexaenoyl-GPC (14:0/22:6)	PC	-0,091	0,061	1,11E-05	Yes
1-myristoyl-2-arachidonoyl-GPC (14:0/20:4)	PC	-0,086	0,061	2,89E-05	Yes
MBOAT7					
**Lipids**					
**Glycerophospholipids**					
1-palmitoyl-2-linoleoyl-GPI (16:0/18:2)*	PI	0,204	0,031	0,00E+00	Yes
1-stearoyl-2-arachidonoyl-GPI (18:0/20:4)*	PI	-0,206	0,031	0,00E+00	Yes
1-stearoyl-2-linoleoyl-GPI (18:0/18:2)*	PI	0,211	0,030	0,00E+00	Yes
1-palmitoyl-2-oleoyl-GPI (16:0/18:1)*	PI	0,184	0,030	0,00E+00	Yes
1-palmitoyl-2-arachidonoyl-GPI (16:0/20:4)*	PI	-0,137	0,029	3,92E-11	Yes
1-stearoyl-2-oleoyl-GPI (18:0/18:1)*	PI	0,118	0,034	1,62E-07	Yes
1-linoleoyl-GPI (18:2)*	Lyso-PI	0,199	0,030	0,00E+00	Yes
1-arachidonoyl-GPI (20:4)*	Lyso-PI	-0,174	0,030	2,00E-17	Yes
1-palmitoleoyl-GPI (16:1)*	Lyso-PI	0,163	0,031	8,02E-14	Yes
1-oleoyl-GPI (18:1)*	Lyso-PI	0,128	0,030	3,71E-10	Yes
GCKR					
**Carbohydrates**					
Mannose*		-0,329	0,028	0,00E+00	No

Pyruvate		0,128	0,029	3,37E-10	No
Lactate		0,100	0,029	9,13E-07	No
**Amino acids**					
threonine	-	-0,093	0,029	4,61E-06	No
isoleucine	-	0,083	0,029	4,41E-05	Yes
1-carboxyethylleucine	Leucine pathway	0,133	0,030	2,20E-09	Yes
1-carboxyethylvaline	Valine pathway	0,125	0,030	5,66E-09	Yes
1-carboxyethylisoleucine	Isoleucine pathway	0,118	0,032	1,66E-07	Yes
gamma-glutamylthreonine	ɣ-glutamyl amino acid	-0,089	0,031	1,26E-05	Yes
gamma-glutamyl-citrulline	ɣ-glutamyl amino acid	-0,086	0,031	2,45E-05	Yes
3-aminoisobutyrate	β-amino acids	-0,095	0,031	3,25E-06	No
4-guanidinobutanoate	ɣ-amino acids	-0,088	0,030	1,56E-05	Yes
**Lipids**					
**Glycerolipids**					
1-myristoylglycerol (14:0)	Monoacylglycerol	0,109	0,031	1,28E-07	No
1-palmitoleoylglycerol (16:1)	Monoacylglycerol	0,105	0,029	3,71E-07	No
1-linolenoylglycerol (18:3)	Monoacylglycerol	0,100	0,031	3,24E-06	Yes
Palmitoyl-oleoyl-glycerol (16:0/18:1) [2]	Diacylglycerol	0,152	0,037	3,47E-09	Yes
Palmitoyl-oleoyl-glycerol (16:0/18:1) [1]	Diacylglycerol	0,193	0,045	5,24E-09	Yes
Oleoyl-arachidonoyl-glycerol (18:1/20:4) [1]	Diacylglycerol	0,114	0,030	5,89E-08	No
Oleoyl-linoleoyl-glycerol (18:1/18:2) [2]	Diacylglycerol	0,109	0,030	1,06E-07	No
Oleoyl-oleoyl-glycerol (18:1/18:1) [1]	Diacylglycerol	0,123	0,034	4,62E-07	No
Oleoyl-oleoyl-glycerol (18:1/18:1) [2]	Diacylglycerol	0,118	0,033	5,12E-07	No

Oleoyl-linoleoyl-glycerol (18:1/18:2) [1]	Diacylglycerol	0,098	0,030	1,92E-06	No
Palmitoleoyl-linoleoyl-glycerol (16:1/18:2) [1]	Diacylglycerol	0,110	0,032	1,94E-06	No
Diacylglycerol (16:1/18:2 [2]; 16:0/18:3 [1]	Diacylglycerol	0,101	0,030	2,76E-06	Yes
Palmitoyl-linoleoyl-glycerol (16:0/18:2) [2]	Diacylglycerol	0,101	0,031	4,85E-06	No
Oleoyl-arachidonoyl-glycerol (18:1/20:4)[2]	Diacylglycerol	0,092	0,031	1,49E-05	No
Myristoyl-linoleoyl-glycerol (14:0/18:2)[2]	Diacylglycerol	0,100	0,035	3,97E-05	Yes
**Glycerophospholipids**					
1-stearoyl-2-oleoyl-GPE (18:0/18:1)	PE	0,107	0,030	1,80E-07	No
1-palmitoyl-2-oleoyl-GPE (16:0/18:1)	PE	0,105	0,029	2,54E-07	No
1-stearoyl-2-docosahexaenoyl-GPE (18:0/22:6)	PE	0,092	0,028	7,54E-06	No
1-stearoyl-2-linoleoyl-GPE (18:0/18:2)	PE	0,089	0,030	1,47E-05	No
1-stearoyl-GPE (18:0)	Lyso-PE	0,097	0,031	2,16E-06	No
1-palmitoyl-GPE (16:0)	Lyso-PE	0,090	0,029	9,97E-06	No
1-oleoyl-GPE (18:1)	Lyso-PE	0,087	0,030	2,03E-05	Yes
1-stearoyl-2-arachidonoyl-GPI (18:0/20:4)	PI	0,109	0,030	9,89E-08	No
1-palmitoyl-2-arachidonoyl-GPI (16:0/20:4)	PI	0,102	0,029	7,17E-07	Yes
1-stearoyl-2-docosapentaenoyl-GPC (18:0/22:5n3)	PC	0,096	0,031	2,69E-06	Yes
1-stearoyl-2-dihomolinolenoyl-GPC (18:0/20:3n3or6)	PC	0,092	0,026	6,61E-06	Yes
1-myristoyl-2-arachidonoyl-GPC (14:0/20:4)	PC	0,090	0,028	1,08E-05	No
1-myristoyl-2-docosahexaenoyl-GPC (14:0/22:6)	PC	0,089	0,028	1,33E-05	Yes
phosphatidylcholine (16:0/22:5n3; 18:1/20:4)	PC	0,086	0,031	2,33E-05	Yes
1-stearoyl-2-docosapentaenoyl-GPC (18:0/22:5n6)	PC	0,085	0,027	3,19E-05	Yes
2-myristoyl-GPC (14:0)	Lyso-PC	0,087	0,029	2,55E-05	Yes
1-myristoyl-GPC (14:0)	Lyso-PC	0,084	0,029	3,81E-05	Yes
1-stearoyl-GPG (18:0)	Lyso-PG	0,099	0,031	1,04E-05	Yes

**Fatty acyls**					
3-methyl-2-oxovalerate	Fatty acid ester	0,091	0,028	7,52E-06	Yes
**Other metabolites**					
Mannonate	-	-0,232	0,030	0,00E+00	Yes
Alpha-ketobutyrate	Keto acids	0,099	0,028	1,14E-06	Yes
Hydroxy-palmitoyl-sphingomyelin (d18:1/16:0 (OH))	-	-0,088	0,031	1,65E-05	Yes
2-hydroxybutyrate/2-hydroxyisobutyrate	α-hydroxy acid	0,086	0,028	2,58E-05	Yes
Glutamine conjugate of C6H10O21	-	-0,086	0,027	2,98E-05	Yes
Glutamine conjugate of C7H12O2	-	-0,088	0,028	3,55E-05	Yes
PPP1R3B					
**Amino acids**					
Glycine*	-	0,138	0,035	1,34E-11	No
Hexanoylglutamine*	-	-0,105	0,037	3,07E-07	Yes
N-acetylkynurenine [2]	N-acyl-L-α-amino acids	-0,098	0,039	2,38E-06	Yes
**Lipids**					
**Glycerophospholipids**					
1-palmitoyl-GPE (16:0)	Lyso-PE	0,087	0,038	1,89E-05	Yes
2-palmitoyl-GPE (16:0)	Lyso-PE	0,085	0,040	3,43E-05	Yes
**Steroids**					
Androstenediol-3-alpha-17-alpha-monosulfate [3]	Sulfated steroids	0,096	0,040	2,53E-06	Yes
Pregnenediol disulfate (C21H34O8S2)	Sulfated steroids	0,092	0,040	6,72E-06	Yes
Pregnenetriol disulfate	Sulfated steroids	0,089	0,039	1,33E-05	Yes
Etiocholanolone glucuronide	Steroid glucuronide conjugates	0,093	0,041	6,49E-06	Yes
**Other metabolites**					

Xanthurenate	Quinoline carboxylic acids	-0,085	0,038	3,64E-05	Yes
HSD17B13					
**Lipids**					
**Glycerophospholipids**					
1-stearoyl-2-linoleoyl-GPE (18:0/18:2)	PE	0,085	0,036	3,37E-05	Yes
1-stearoyl-2-oleoyl-GPE (18:0/18:1)	PE	0,085	0,036	3,49E-05	Yes

Abbreviations: Lyso-PC, lysophosphatidylcholine; lyso-PE, lyso-phosphatidylethanolamine; lyso-PG, lyso-phosphatidylglycerol; lyso-PI, lyso-phosphatidylinositol; PC, phosphatidylcholine; PE, phosphatidylethanolamine; PI, phosphatidylinositol;

*No statistically significant differences in the betas from linear regression analysis between the carriers of the same genetic variants with and without NAFLD.
